# Whole brain radiotherapy with a conformational external beam radiation boost for lung cancer patients with 1-3 brain metastasis: a multi institutional study

**DOI:** 10.1186/1748-717X-5-13

**Published:** 2010-02-18

**Authors:** Nathalie Casanova, Zohra Mazouni, Sabine Bieri, Christophe Combescure, Alessia Pica, Damien C Weber

**Affiliations:** 1 Radiation Oncology, Geneva University Hospital, 6 rue Gabrielle Perret Gentil, CH-1211 Geneva, Switzerland; 2 Radiation Oncology, Centre Hospitalier Universitaire Vaudois, Rue du Bugnon 21, CH-1001 Lausanne, Switzerland; 3 Radiation Oncology, Sion Cantonal Hospital, Av. du Grand-Champsec 80, CH-1950 Sion, Switzerland; 4 Clinical Epidemiology Unit, Geneva University Hospital, 6 rue Gabrielle Perret Gentil, CH-1211 Geneva, Switzerland; 5 University of Geneva, 1 rue Michel Servet, CH-1205 Geneva, Switzerland

## Abstract

**Background:**

To determine the outcome of patients with brain metastasis (BM) from lung cancer treated with an external beam radiotherapy boost (RTB) after whole brain radiotherapy (WBRT).

**Methods:**

A total of 53 BM patients with lung cancer were treated sequentially with WBRT and RTB between 1996 and 2008 according to our institutional protocol. Mean age was 58.8 years. The median KPS was 90. Median recursive partitioning analysis (RPA) and graded prognostic assessment (GPA) grouping were 2 and 2.5, respectively. Surgery was performed on 38 (71%) patients. The median number of BM was 1 (range, 1-3). Median WBRT and RTB combined dose was 39 Gy (range, 37.5 - 54). Median follow-up was 12.0 months.

**Results:**

During the period of follow-up, 37 (70%) patients died. The median overall survival (OS) was 14.5 months. Only 13 patients failed in the brain. The majority of patients (*n *= 29) failed distantly. The 1-year OS, -local control, extracranial failure rates were 61.2%, 75.2% and 60.8%, respectively. On univariate analysis, improved OS was found to be significantly associated with total dose (≤ 39 Gy *vs*. > 39 Gy; p < 0.01), age < 65 (p < 0.01), absence of extracranial metastasis (p < 0.01), GPA ≥ 2.5 (p = 0.01), KPS ≥ 90 (p = 0.01), and RPA < 2 (p = 0.04). On multivariate analysis, total dose (p < 0.01) and the absence of extracranial metastasis (p = 0.03) retained statistical significance.

**Conclusions:**

The majority of lung cancer patients treated with WBRT and RTB progressed extracranially. There might be a subgroup of younger patients with good performance status and no extracranial disease who may benefit from dose escalation after WBRT to the metastatic site.

## Background

Brain metastases (BMs) occur in up to 40% of all adult cancer patients[1], and are the most frequent type of brain malignancy. They represent usually a late event during the course of the malignancy. Up to 200,000 new cases per year are newly diagnosed in North America[2]. The incidence of BM may have increased, possibly as a paradoxical result of the effectiveness of anti-cancer drugs that do not cross the blood-brain barrier, but acts effectively on the primary tumour and/or extracranial metastases[3]. Alternatively, improved diagnostic strategies[4] or clonal selection[5] could also explain the observed increase of BM incidence. As such, BMs represent a major complication of cancer patient's survivorship.

Most BMs originate from the lung (40-50%), breast (15-25%), melanoma (5-20%) or kidney (5-10%)[1]. Even after whole brain radiotherapy (WBRT), the prognosis of BM patients is poor, with a reported median overall survival (OS) of 2.5 to > 6.0 months [6-8] and may be somewhat overestimated by the patient and referring physician alike[9].

WBRT, when compared to best supportive care only, increases significantly OS. WBRT results, more often than not, in a worthwhile, albeit temporary, improvement in the patient's medical condition. In a multicentric prospective phase III trial, the 3-months radiological response rate, assessed by central review, was 70% after WBRT[10]. Nevertheless, the prognosis of these BM patients remains dismal, as they fail locally in substantial number cases. In the RTOG 9508 trial, the observed 1-year local failure rate was approximately 30%[10]. In another phase III study, the 1-year brain failure rate was as high as 100%[11]. As such, decreasing the local tumour failure rate after WBRT is desirable in BM patients. It has been recently shown that brain recurrence had a major impact on the patient's neuro-cognitive function[12] and thus quality of life (QoL)[13].

For multiple BMs, several retrospective [14-17] and prospective[18,19] historical studies have assessed the influence of dose on outcome but none of these studies have shown a survival advantage for high doses. Two prospective randomized trials have however shown that adjuvant radiosurgery increased significantly the brain control rate in patients with a limited number of BMs[10,11].

In this Swiss multicenter retrospective study we assessed the outcome and pattern of failures in lung cancer patient presenting 1 to 3 BM treated sequentially with WBRT and external beam radiotherapy boost (RTB).

## Methods

### Patients

Cases were identified in the radiation oncology departments of Geneva University Hospital (HUG), Sion Cantonal Hospital (CHCVS) and the University Hospital of Lausanne (CHUV) databases. All three institutions shared a common therapeutic protocol for BM patients. The inclusion criteria for this retrospective analysis were: 1) patients with 1 - 3 brain metastasis; 2) KPS ≥ 50; 3) age ≤ 80 years; 4) No previous radiotherapy to the brain; 5) WBRT and 6) conformational boost using external beam RT. No histopathology of the brain lesion was required but a pathological diagnosis of cancer for the primary tumour was necessary. Eighty three of such patients were identified. Only patients with a primary lung cancer tumour were retained for this analysis. As such, a cohort of 53 patients is the basis of the analysis, treated between May 1996 and November 2008 in the three institutions. The patient's characteristics are detailed in Table 1. No significant patient characteristics' differences were observed when stratified by centers, except for dose and lung cancer type (Table 1). Sixteen (30%) and 37 (70%) patients presented with and without extracranial disease, respectively. KPS ranged from 50 to 100 (median, 90). All patients were classified prospectively using the KPS performance and RPA prognostic[20] scales in the institutional databases and retrospectively using the GPA prognostic scale[21] for the purpose of this study.

**Table 1:** Patient characteristics (*n *= 53)

Variable	CHUV	Number (%) HUG	CHCVS	p*
**Age **(years)				0.68
Median	57	61	55	
Range	48 - 73	41 - 76	25 - 78	

**Gender**				0.99
Female	6 (46)	7 (26)	5 (39)	
Male	7 (54)	20 (74)	8 (61)	

**GPA**				0.50
Median	3.0	2.5	2.5	
Range	2 - 4	1 - 4	1 - 4	

**RPA**				0.28
1	5 (38)	8 (30)	6 (46)	
2	8 (62)	12 (44)	5 (39)	
3	0 (0)	7 (26)	2 (15.4)	

**Lung cancer, type**				0.03
Adenocarcinoma	9 (69)	17 (63)	6 (46)	
SCC	1 (23)	4 (22)	7 (54)	
Neuro-endocrine	3 (8)	6 (15)	0 (0)	

**Number of metastasis**				0.88
1	12 (92)	22 (82)	11 (85)	
2 - 3	1 (8)	5 (18)	2 (15)	

**Brain metastasis**				0.13
Synchronous	21 (78)	6 (46)	8 (62)	
Metachronous	6 (22)	7 (54)	5 (38)	

**Brain surgery **(metastatectomy)				0.44
Yes	11 (85)	19 (70)	8 (62)	
No	2 (15)	8 (30)	5 (38)	

Dose (Gy)				< 0.01
≤ 39	0	26	3	
> 39	13	1	10	

* Fisher test, except for age and GPA (Kruskal-Wallis test)

### Treatment

Surgery was performed in 38 (72%) patients (gross total excision, *n *= 36; partial excision, *n *= 2; Table 1). WBRT was administered using megavoltage photons with two lateral fields. Median dose of WBRT was 25 Gy (range, 25 - 45). The WBRT dose per fraction ranged from 1.8 to 3 Gy (median, 3). After WBRT, a boost to the metastatic site was administered with external beam radiotherapy. Stereotactic radiotherapy was not delivered for RTB. Virtual simulation was used for RTB planning, with a median margin of 10 mm (range, 10 - 25) around the metastasis/metastases, in all patients. Median boost dose was 9 Gy (range, 7.5 - 18). The RTB dose per fraction ranged from 1.8 to 3 Gy (median, 3). The median total dose administered to the metastatic sites was 39 Gy (range, 34.5 - 54).

### Follow-up evaluation

Follow-up was obtained by office visit in the authors (SB, AP and DCW) clinics, correspondence with the referring physician or by direct telephone contact with patients. Serial brain imaging studies (MRI or contrast-enhanced CT) were requested usually before or after the clinical follow-up, or if the patient presented with clinical progressive disease (PD). All side effects seen after 90 days from the end of radiotherapy were considered late adverse events. These were classified according to the National Cancer Institute Common Terminology Criteria for Adverse Events (CTCAE), ver. 3.0 grading system http://ctep.cancer.gov.

### Statistical analysis

Local control (LC), extracranial failure (ECF), progression-free survival (PFS) and overall survival (OS) rates at 1 year were calculated from the date of WBRT using Kaplan Meier estimates. Recorded events were the absence of local failure at the metastatic brain site and PD at non-CNS sites for LC and ECF, respectively, or death, local, brain or extra cranial failure or death for PFS and death (all causes of death included) for OS. PD was defined as any increase in tumour size or recurrent tumour either at the metastatic brain site, in the brain or extracranially. The association between the factors and the mortality and the relapse was explored by univariate and multivariate survival analyses. In the univariate survival analysis, the survival curves were assessed by using the Kaplan-Meier's estimator and compared with the log rank's test. In the multivariate analysis, a Cox regression model was used and the hazard ratios are reported with the 95% confidence intervals. The variables with a p-value less than 0.10 were introduced in the Cox model, and a selection procedure was performed. We checked that the selected variables were the same by either forward or backward procedure. Only the final model was reported. Statistical tests were based on a two-sided significance level, and a *p *value of 0.05 or less was considered statistically significant. The statistical analysis was performed on the Statistical Package for Social Sciences system (SPSS, Ver.17.0, SPSS Inc., Chicago, IL).

## Results

**Figure 1:**
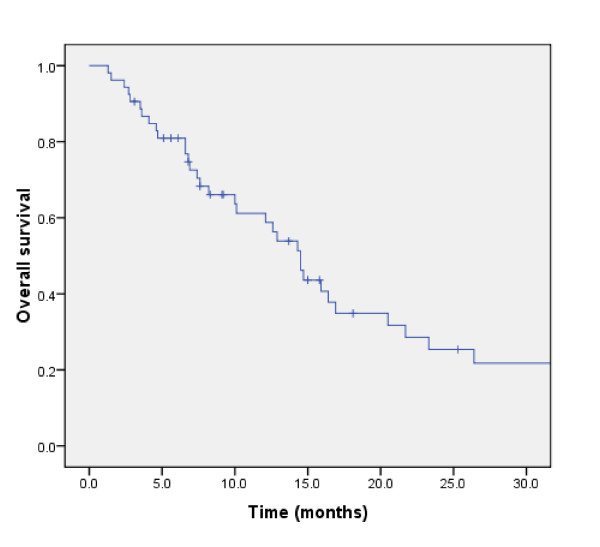
**Overall survival in 53 lung cancer patients treated with WBRT and RTB**.

After a median follow-up of 12.0 months (range, 3.0 - 56.0), 37 (70%) patients died. The median OS was 14.5 ± 1.3 months. The 6 month- and 1-year actuarial OS rates were 80.9% and 61.2%, respectively (Fig. 1). Cause of death was PD in a majority of patients (*n *= 33; 89.2%). Among these 33 PD patients, 25 and 8 died of extracranial and brain progression, respectively. Three (8.1%) patient died of bronchopneumonia. Postoperative death for second Head & Neck cancer was observed in (2.7%) another patient.

Overall, 38 disease progression were observed. The median time to disease progression was 7.3 ± 1.1 months. The 6 month- and 1-year PFS rates were 62.9% and 26.7%, respectively. The majority of patients with PD presented with extra cranial PD. Eighteen (47.4%) patients failed extracranially as the sole side of PD, 14 (36.8%) failed in the brain only and 6 (15.8%) progressed at the metastatic brain site only.

**Figure 2:**
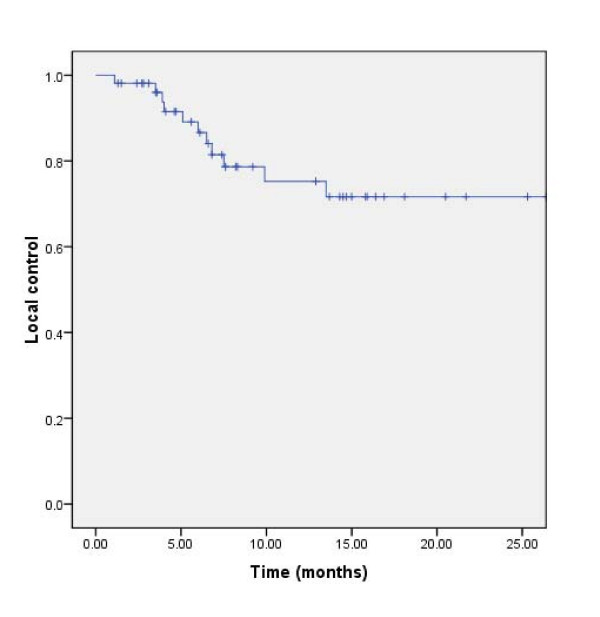
**Local control in 53 lung cancer patients treated with WBRT and RTB**.

Overall, local failure was observed in (24.5%) 13 patients (Fig. 2). The median time to local failure was 48.9 ± 11.5 months. The 6 month- and 1-year local control rates were 98.1% and 75.2%, respectively. Local failure only was observed in 6 patients and another 7 patients presented local brain failure with concomitant distant brain failure.

Distant brain failure was observed in 14 (26.4%) patients. The median time to distant brain failure only was 48.9 ± 25.1 months. The 6 month- and 1-year brain failure rates were 10.8% and 28.2%, respectively. Brain failure only was observed in 7 patients and another 7 patients presented local brain failure with concomitant distant brain failure.

Extra cranial failure was observed in 29 (54.7%) patients. Median time to extra cranial failure was 10.4 ± 1.1 months. The 6 month- and 1-year local control rates were 29.5% and 60.8%, respectively. Extra cranial failure only was observed in 18 patients, 6 and 3 patients presented with extra cranial failure/local brain failure/distant brain failure and extra cranial failure/distant brain failure, respectively. Extra cranial failure and local brain failure only was observed in another 2 patients.

Late radiation-induced toxicity was minimal: alopecia (grade CTCAE 1, 15 and grade CTCAE 2, 3 patients) was observed in 18 (33.9%) patients. No patient presented with gross neuro-cognitive dysfunction. Asthenia grade CTCAE grade 1 and 2 was observed in 11 patients, respectively. No patient presented with asthenia CTCAE grade 3.

**Figure 3:**
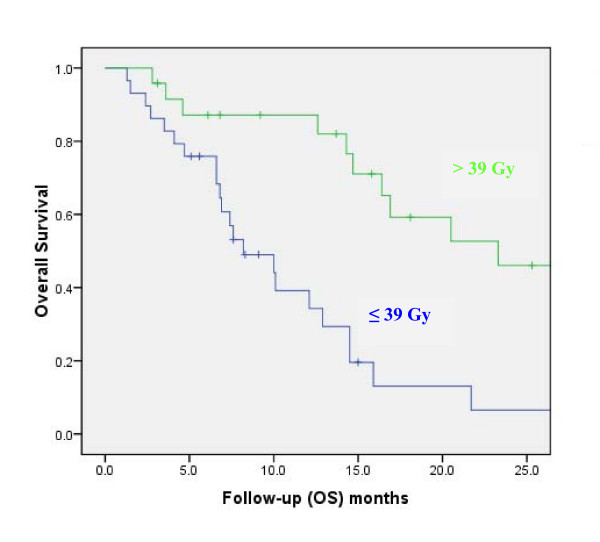
**Overall survival (OS) by RT dose group for 53 BM patients with lung cancer**.

On univariate analysis (Table 2), improved OS was found to be significantly associated with total dose (≤ 39 Gy *vs*. > 39 Gy; p < 0.01; Fig. 3), age < 65 (p < 0.01), absence of extracranial metastasis (p < 0.01), GPA ≥ 2.5 (p = 0.01), KPS ≥ 90 (p = 0.01), and RPA = 2 (p = 0.02). Gender was not found to be associated with survival but there was a trend for statistical significance of improved OS in patients female *vs*. patients male (p = 0.07; Table 2). Likewise, there was a statistical trend toward significance for surgery (p = 0.07; Table 2) and center (p = 0.07; Table 2). The number of brain metastasis (p = 0.49; Table 2), histology (p = 0.58; Table 2) and synchronous *vs*. metachronous (p = 0.71) were however not found to be associated significantly with survival. On multivariate analysis, only total dose (hazard ratio [HR], 3.55; 95% confidence interval [95%CI], 1.65 - 7.64; p < 0.01) and the absence of extracranial metastasis (HR 2.29; 95%CI, 1.10 - 4.73; p = 0.03) retained statistical significance.

**Table 2:** Summary of univariate anlaysis for OS and PFS

	median OS (months)	p* (HR [95%])	median PFS (months)	p*
Age, years				
< 65	15.9	<0.01	9.3	<0.01
≥ 65	7.4	(3.75 [1.51-9.31])	3.8	(3.10 [1.44-6.69])

Total dose, Gy				
≤ 39	8.2	<0.01	3.9	<0.01
> 39	23.3	(3.84 [1.83-8.03])	11.7	(0.29 [0.14-0.59])

GPA				
≥ 2.5	15.9	0.01	9.3	0.01
< 2.5	7.4	(2.42 [1.18-4.93])	3.8	(2.46 [1.23-4.92])

Extracranial metastasis				
Yes	7.6	<0.01	3.8	<0.01
No	16.9	(2.71 [1.35-5.44])	9.3	(2.93 [1.48-5.79])

KPS				
≥ 90	14.7	0.01	5.1	0.05
< 90	7.6	(2.35 [1.19-4.63])	9.3	(2.26 [0.98-5.22])

RPA				
1	14.7	0.02	8.3	0.01
2-3	7.6	(2.46 [1.10-5.50])	3.8	(2.72 [1.25-5.89])

Gender				
Female	16.4	0.07	7.4	0.58
Male	12.6	(1.94 [0.93-4.03])	6.2	(1.21 [0.61-2.41])

Center				
CHUV	26.4	0.07	33.3	0.05
HUG	10.1	(2.76 [1.12-6.80])	4.1	(3.23 [1.21-8.65])
CHCVS	16.4		9.0	

Surgery				
Yes	7.5	0.07	3.8	0.05
No	15.9	(1.90 [0.94-3.82])	9.3	(0.51 [0.25-1.01])

Number of brain metastasis				
1	16.4	0.49	7.4	0.61
2-3	14.3	(0.72 [0.28-1.86])	6.6	(0.78 [0.30-2.02])

Type of primary ling cancer				
SCC	14.5	0.58	6.2	0.40
AdenoCa	14.7	(1.61 [0.64-4.02])	9.3	(1.67 [0.70-3.99])
Neuroendocrine	12.6	(1.61 [0.64-4.02])	6.5	

*log-rank

Improved PFS was found to be significantly associated with age < 65 (p < 0.01), total dose (≤ 39 Gy *vs*. > 39 Gy; p < 0.01), absence of extracranial metastasis (p < 0.01), RPA < 2 (p = 0.01), GPA ≥ 2.5 (p = 0.01), T stage (p = 0.02), metachronous *vs*. synchronous BM (p = 0.03), N stage (p = 0.05), KPS ≥ 90 (p = 0.05) and center (p = 0.05). On multivariate analysis, total dose (HR 3.63; 95% CI 1.60 - 8.24; p < 0.01), T stage (HR 3.02; 95% CI 1.32 - 6.89; p < 0.01), and the absence of extracranial metastasis (HR 5.79; 95% CI 2.52 - 13.32; p < 0.01) retained statistical significance.

## Discussion

To the best of our knowledge, the present study is the largest series ever published on WBRT with RTB in the treatment of lung cancer patients with BM. The observed progression disease pattern was mainly extracranially, with 3 patients out of 4 with disease progression deceasing from systemic disease. As such, the estimated LC rate was remarkable, with a 1-year LC rate of more than 75% (Fig. 2)

The significant influence of total dose on duration of survival in this cohort of patients with metastatic lung cancer was the main finding of this analysis (Fig. 3). The addition of a RTB to WBRT appeared to substantially increase the median OS to approximately 15 months (Fig. 1), which compares favourably with those of other series of radiosurgery (SRS), with[22] or without surgery [10,11,23] or concomitant targeted agent[24]. A survival advantage of SRS to WBRT in patients with multiple BMs was not observed in the RTOG 9805 study randomising 333 patients with 1 to 4 BM[10]. The mean OS was 6.5 and 5.7 months (p = 0.13) in the WBRT alone and combined modality arms, respectively. Patients with single BM treated with adjuvant SRS had however a significant better survival (4.9 *vs*. 6.5 months; p = 0.04) than those who were not allocated boost treatment. Likewise, a smaller prospective trial randomising 27 patients with 1 - 4 BM to WBRT ± SRS did not show a significant increase in survival (7.5 *vs*. 11.0 months, p = 0.22)[11].

The influence of RTB (15 Gy in 8 fractions) was also assessed in 50 BM patients treated with 30 - 40 Gy WBRT[25]. The mean OS of these patients was 4.6 months, compared to 3.8 months for those (*n *= 114) receiving WBRT alone. Hoskin *et al*. concluded that no advantage of high dose adjuvant radiation treatment could be foreseen using external beam radiotherapy. Approximately 60% of patients with a single BM received RTB in this study on the basis of stable disease and good general condition. Possible explanations for this discrepant finding include imbalances between the two cohorts with respect to known and unknown baseline prognostic factors (no prognostication was possible for the Royal Marsden Hospital study) or imbalances in the use of second and third-line therapies, as the majority of patients (60% - 75%) died of metastatic disease outside the brain in both studies. Our results are however in line with the retrospective analysis of 201 patients with 1 - 2 BMs[1]. All patients were RPA 1 or 2 and they underwent resection of the metastasis and WBRT with (*n *= 102) or without (*n *= 99) a RTB. The median OS was 18 and 9.5 months (p < 0.001) for the former and latter group, respectively. On multivariate analysis, RTB, extent of surgical resection and interval from the tumour diagnosis and RT were found to be statistically significant. Interestingly, the median OS observed in our study, constituted of a majority (>70%) of patients undergoing surgery, is identical (14.5 months) to the one reported by the German group. The addition of a RTB was also associated with improved local tumour and brain control[1]. Noteworthy, increasing the dose to the surgical bed with 10 - 15 Gy RTB after WBRT did not modify the patient outcome in a recent match-pair analysis with patient treated with WBRT and radiosurgery[26].

The present study evaluated 11 prognostic factors for OS and PFS. An administered dose of > 39 Gy was associated with a significant increase in OS and PFS (Table 2). Interestingly, the parameter center was associated with a significant improvement in patient outcome in univariate analysis (Table 2). One center did always administer sequentially 36 Gy with WBRT and 18 Gy with RTB (Table 1). As dose was a significant prognosticator, this factor did not retain significance in the multivariate analysis. Assuming a α-β ratio of 10 for lung cancer, the 54 Gy (delivered in 2 Gy per fraction) and 39 Gy (delivered in 3 Gy per fraction) will correspond to a biological effective dose (BED) of 65 and 51 Gy_10_, respectively. The magnitude of the >25% increase in BED might be expected to result in an increase in LC for BM patients treated with the former dose schedule. This strategy will however consequentially translate in an increase of the overall treatment time that could be detrimental for poor prognosis patients with a limited OS. The other significant prognostic factor for OS and PFS was the absence of extra cranial disease, which is a recognized prognosticator for BM patients undergoing RT[20].

We could not assess the long term neuro-cognitive effect of this RTB strategy, as only one center prospectively performed Mini Mental Status Examination in all BM patients. The patients treated in this center had however the lower survival rate, so we had unfortunately insufficient baseline and follow-up data to adequately assess neuro-cognition. We were however unaware of any such toxicity in patients who were followed in our respective clinics. The observed >75% of LC could possibly result in an increase of neuro-cognitive function for our patients treated with WBRT and RTB. Regine *et al*. reported on the neuro-cognitive outcome of 445 BM patients treated in the RTOG 91-04 phase III study[27]. Control of BM had a significant impact on neuro-cognition as measured by the Mini-Mental Status Examination. Likewise, Meyers *et al*. reported on another phase III trial assessing the efficacy of gadolinium motexafin[12]. Patients with BM from lung cancer presented with an increase of fine motor and visual motor scanning function if they had a partial response on brain MRI. All patient with PD had a decline of neuro-cognitive function.

It is appropriate to acknowledge that, in a retrospective analysis spanning more than 12 years, the apparent striking impact of total dose on outcome might be at least partially reflect confounding factors. RTB was delivered only to patients with a good prognosis and, as such, this treatment policy should not be delivered indiscriminately to all BM patients. The majority of patient underwent surgical resection, but 15% of the cohort did not benefit from surgery. The patients treated in one center delivering high dose RT did present a more favourable prognostic profile, although not significantly so (Table 1). It should be noted however that there was no difference in age, number of BM or percentage of operated patients (Table 1). We were thus unable to identify other factors that might adequately explain the observed effect. There was another limitation to our study. The small sample size of 53 patients and its consequential statistical power limits the overall conclusions of this study. We have chosen to perform however a multivariate analysis, as the ratio of observations to prognostic factors was appropriate[28]. Further research regarding RT dose-outcome relationships is justified in the framework of modern technique delivery.

## Conclusions

This analysis of the outcome of 53 lung cancer patients with BM treated with WBRT and RTB reveals an increase in OS and PFS for patients treated with higher radiation doses. Only one-quarter of the studied cohort presented with local failure. The majority of patients presented with extra cranial progression. There might be a subgroup of younger patients with good performance status and no extracranial disease who may benefit from non-stereotactic dose escalation after WBRT to the metastatic site.

## Abbreviations

BM: brain metastasis; RTB: radiotherapy boost; WBRT: whole brain radiation therapy; QoL: quality of life; MRI: magnetic resonance imagery; CT: computed tomography; PD: progressive disease; CTCAE: Common Terminology Criteria for Adverse Events; LC: local control; ECF: extracranial failure; OS: overall survival; PFS: progression-free survival; KPS: Karnofsky performance status; RPA: recursive partitioning analysis; GPA: graded prognostic assessment; SCC: Squamous cell carcinoma; BED: biologic effective dose.

## Competing interests

The authors declare that they have no competing interests.

## Authors' contributions

DCW was responsible for the primary concept and the design of the study; DCW, NC, ZM and SB performed the data capture and analysis. NC and DCW drafted the manuscript; DCW and CC performed the statistical analysis; DCW, NC, ZM and SB reviewed patient data; AP, SB, CC and MZ revised the manuscript.

All authors have read and approved the final manuscript.
